# Macrophage Phenotypic Switch and Obesity-Associated Metabolic Risk: Mechanisms and Targets

**DOI:** 10.1155/omcl/6710641

**Published:** 2025-11-16

**Authors:** K. F. Hinojosa Vera, C. Hemakumar, R. S. Bilachi, D. C. Ramirez, S. E. Gomez Mejiba

**Affiliations:** ^1^Laboratory of Experimental and Translational Medicine, Institute Multidisciplinary of Biological Research, CONICET-SL. National University of San Luis, San Luis, Argentina; ^2^Department of Biotechnology, Dayananda Sagar College of Engineering, Bangalore, Karnataka, India; ^3^Department of Biotechnology, Manipal Institute of Technology, Manipal Academy of Higher Education, Manipal, Karnataka, India; ^4^Laboratory of Nutrition and Experimental Therapeutics, Institute Multidisciplinary of Biological Research, CONICET-SL. National University of San Luis, San Luis, Argentina

**Keywords:** adipose tissue, inflammation, macrophage, metabolic risk, microenvironment, obesity, polarization

## Abstract

Obesity-associated metabolic dysfunction is closely linked to chronic low-grade inflammation, or metaflammation, which is predominantly driven by changes in AT homeostasis. Macrophages, key components of the innate immune system, are central regulators of this inflammatory process. In lean AT, resident macrophages (AT-associated macrophages [ATMs]) exhibit an anti-inflammatory phenotype and support tissue homeostasis. However, during obesity, AT undergoes hypoxia, mechanical stress, and lipid overload, leading to immune cell infiltration and a phenotypic switch of ATMs toward a proinflammatory M1 profile. This shift contributes to systemic inflammation and obesity-associated metabolic risks. Here, we review the current understanding of macrophage polarization in obesity, highlighting the transcriptomic plasticity and functional heterogeneity of ATMs, their interactions within the AT microenvironment, and the formation of crown-like structures (CLSs) as a structural hallmark of AT inflammation. We also discuss the regulatory functions of transcription factors, such as hypoxia-inducible factor (HIF) 1α (HIF-1α) and peroxisome proliferator activated receptor gamma (PPARγ), that control the phenotypic switch of macrophages in healthy and obese ATs. Furthermore, we examined emerging macrophage subsets, such as CD9^+^ and Trem2^+^ lipid-associated macrophages (LAMs), and their dual roles in AT remodeling and inflammation. Understanding the complex network of macrophage activation in obese AT is essential for identifying therapeutic targets aimed at mitigating obesity-associated metabolic risk and restoring tissue function.

## 1. Introduction

Macrophages are cells of the innate immune system that are characterized by the detection, phagocytosis, and destruction of bacteria and/or other invading microorganisms [1]. These cells are involved in the presentation of antigens for the development of the adaptive immune response and can secrete cytokines and chemokines to initiate the inflammatory process against injury [2].

Macrophage phenotypes can be divided into two main subtypes: M1-type macrophages and M2-type macrophages [3]. M1 macrophages are described as the proinflammatory type and are important for direct host defense against pathogens, including phagocytosis and the secretion of proinflammatory cytokines and microbicidal molecules [4]. M2 macrophages have opposite functions, regulating the resolution phase of inflammation and repairing damaged tissues [5]. More recent in vitro and larger ex vivo studies have shown that macrophage phenotypes are much more diverse, overlapping with one another in terms of gene expression and function, revealing that these many hybrid states form a continuum of activation states that depend on the microenvironment [6]. There is great diversity in the gene expression profiles of different populations of tissue macrophages, which is why the activation spectrum of macrophages is considered broader and involves a complex regulatory pathway that responds to many different signals from the adipose tissue microenvironment [7, 8].

Systemic inflammation plays a crucial role in the development of metabolic risks associated with obesity [9]. Chronic low-grade inflammation, often referred to as “metaflammation,” is a hallmark of obesity resulting from the expansion of AT [10]. This expansion leads to hypoxia, mechanical stress on adipocytes, and the release of proinflammatory cytokines [11]. These factors collectively trigger an immune response that recruits inflammatory cells such as macrophages and T cells into the AT, perpetuating the inflammatory state [12]. This systemic inflammation exacerbates obesity-associated metabolic risk, such as insulin resistance (IR), type 2 diabetes (T2D), and cardiovascular disease (CVD) [13]. Recent research highlights that the interplay between metabolic dysregulation and immune activation is central to the pathogenesis of these obesity-related conditions, underscoring the importance of targeting the macrophage phenotypic switch and consequent AT inflammation in therapeutic strategies for reducing obesity-associated metabolic risk [14].

In this review, we summarize the most recent findings concerning AT inflammation and obesity, highlighting the physiological, cellular, and molecular mechanisms through which macrophage phenotype switching and subsequent AT inflammation contribute to AT dysfunction, systemic inflammation, and obesity-associated metabolic abnormalities.

## 2. Adipose Tissue Macrophages: Origin and Polarization

**Figure 1:**
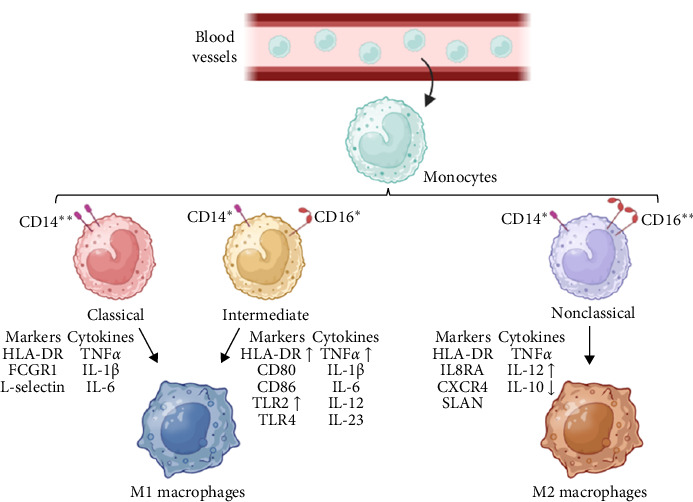
Monocyte differentiation and polarization. Differentiation of circulating monocytes into three subsets: classical (CD14^++^, CD16^−^), intermediate (CD14^+^, CD16^+^), and nonclassical (CD14^+^, CD16^++^) monocytes. Classical and intermediate monocytes can differentiate into M1 proinflammatory macrophages, whereas nonclassical monocytes can differentiate into M2 anti-inflammatory macrophages. CD68, macrophage antigen CD68; CD80, CD80 antigen; CXCR4, C-X-C motif chemokine receptor 4; FCGR1, Fc gamma receptor 1; HLA-DR, major histocompatibility complex class II antigen DR; IL-1β, interleukin-1 beta; IL-6, interleukin-6; IL8RA, interleukin-8 receptor A; IL-10, interleukin-10; IL-12, interleukin-12; IL-23, interleukin-23; SLAN, selenocysteine insertion sequence-binding protein 2-like; TLR2, toll-like receptor 2; TLR4, toll-like receptor 4; TNFα, tumor necrosis factor alpha.

Circulating monocytes in the blood come from the bone marrow through a process known as hematopoiesis, where hematopoietic stem cells differentiate into myeloid progenitors. In response to growth factors such as granulocyte–macrophage colony stimulating factor (GM-CSF) or granulocyte–colony stimulating factor (G-CSF), these cells generate granulomonocytic progenitors and then monoblasts, which in turn also differentiate into premonocytes in the presence of these growth factors; thus, they ultimately differentiate into mature monocytes, which leave the bone marrow and enter the bloodstream [15]. There are different populations of monocytes in circulation; classical monocytes represent 85% of circulating monocytes and present CD14^++^ and CD16^−^ as surface markers; intermediate monocytes represent 5% of monocytes in the blood and have CD14^+^ and CD16^+^ surface markers; and nonclassical monocytes present CD14^+^ and CD16^++^ surface markers and constitute the remaining 10% of circulating monocytes (Figure 1) [16, 17]. These immune cells migrate to peripheral tissues in response to some stimulus; within these tissues, they differentiate into macrophages or dendritic cells and trigger specific responses owing to the pressure of the tissue microenvironment in which they are found; these responses occur owing to the phenotypic heterogeneity presented by macrophages owing to the previous polarization of monocytes [1, 3].

Macrophage polarization is a process by which macrophages produce different functional programs in response to microenvironment signals [5]. Macrophage phenotypes have multiple functions in the body: powerful effector cells of the innate immune system, removal of cellular waste, tissue homeostasis and repair, and embryonic development [18]. Macrophages can be polarized into the M1 and M2 phenotypes, so they perform different functions under certain conditions of the cellular microenvironment; these phenotypes differ in their transcriptomics, proteomics and biological functions owing to their surface markers and secreted cytokines [16].

### 2.1. M1 Macrophage Phenotype

The M1 macrophage phenotype is activated by the “classical pathway” through the recognition of pathogen-associated molecular patterns (PAMPs), such as lipopolysaccharide (LPS); inflammatory cytokines, such as interferon gamma (IFNγ) and tumor necrosis factor alpha (TNFα); and reactive oxygen species (ROS) and reactive nitrogen species (RNS) [16]. The surface markers expressed in this macrophage phenotype are CD80 antigen (CD80), CD86 antigen (CD86), toll-like receptor 2 (TLR-2), toll-like receptor 4 (TLR-4), major histocompatibility complex class II (HLA-DR), and IFNγ receptor (IFNγR) [19].

**Figure 2:**
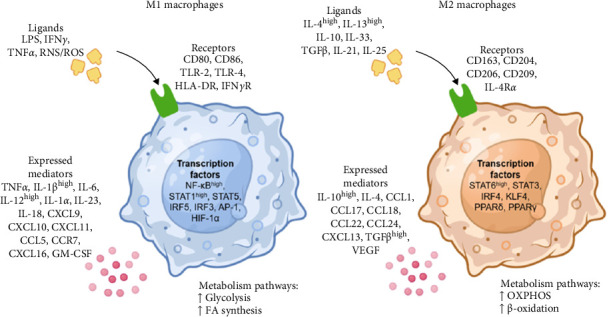
Macrophage polarization. The different stimuli or ligands, receptors, transcription factors, and metabolism pathways of M1 and M2 macrophages are summarized.

M1 macrophages are generated upon stimulation by proinflammatory signals that trigger intracellular signaling cascades, ultimately activating key transcription factors. These include nuclear factor kappa-light-chain-enhancer of activated B cells (NF-κB) and signal transducer and activator of transcription 1 (STAT1), which are strongly associated with the classical activation pathway. Additionally, other transcription factors, such as STAT5, the interferon regulatory factors IRF5 and IRF3, activator protein-1 (AP-1), and hypoxia-inducible factor (HIF) 1 α (HIF-1α), contribute to shaping the M1 phenotype [2, 7]. The activation of these transcription factors enhances the inflammatory, antimicrobial, and antitumor functions of M1 macrophages. This is achieved through the secretion of high concentrations of proinflammatory cytokines, including TNFα, interleukin-1 beta (IL-1β), IL-6, IL-12, IL-1 α (IL-1α), IL-23, and IL-18. Furthermore, they produce chemokines such as CXCL9, CXCL10, CXCL11, CCL5, CXCL16, and GM-CSF, as well as surface molecules such as CCR7, which promote immune cell recruitment (Figure 2). While these mediators are essential for host defense, their prolonged expression can drive chronic inflammation and contribute to disease pathogenesis [20, 21].

In addition, to increase their ability to eliminate pathogens, M1 macrophages produce greater amounts of radical nitric oxide (^·^NO) and citrulline from arginine, products of inducible nitric oxide synthase (iNOS) pathway metabolism [22]. Owing to their ability to fight pathogens, M1 macrophages are present during acute infectious diseases [23]. Several studies have shown that bacterial infection, the early stage of inflammation and chronic inflammatory processes induce macrophage polarization toward the M1 phenotype, resulting in phagocytosis and the intracellular killing of bacteria as well as the removal of damaged or dead body cells [23–25]. Inadequate control of the inflammatory response triggered by M1 macrophages can lead to alterations in tissue homeostasis and prevent injury repair [26].

### 2.2. M2 Macrophage Phenotype

The M2 macrophage phenotype is activated by the “alternative or nonclassical pathway,” also known as anti-inflammatory macrophages; activation is produced by high levels of interleukin-4 (IL-4) and interleukin-13 (IL-13) and other ligands, such as IL-10, IL-33, transforming growth factor beta (TGFβ), IL-21, and IL-25 [22]. The surface markers expressed in this macrophage phenotype are macrophage-associated antigen (CD163), macrophage scavenger receptor 1 (CD204), mannose receptor type-1 (CD206), CD209 antigen (CD209), and IL-4 receptor alpha (IL-4Rα) [2, 22].

The activation of M2 macrophages triggers the regulation of signaling pathways that produce the following transcription factors: signal transducer and activator of transcription 6 (STAT6), signal transducer and activator of transcription 3 (STAT3), interferon regulatory factor 4 (IRF4), Krüppel-like factor 4 (KLF4), peroxisome proliferator activated receptor delta (PPARδ), and peroxisome proliferator activated receptor gamma (PPARγ) [20, 27]. M2-polarized macrophages secrete interleukins such as IL-10 and IL-4; the chemokines C–C motif chemokine ligand 1 (CCL1), C–C motif chemokine ligand 17 (CCL17), C–C motif chemokine ligand 18 (CCL18), C–C motif chemokine ligand 22 (CCL22), C–C motif chemokine ligand 24 (CCL24), and C–X–C motif chemokine ligand 13 (CXCL13); the growth factors TGFβ and vascular endothelial growth factor (VEGF); and other components involved in humoral immunity, wound healing and tolerance of autoantigens and neoantigens [28–30]. Thus, M2 macrophages govern functions at the interfaces of immunity, infection prevention, tissue development, removal and repair, angiogenesis, and immunomodulation (Figure 2) [16, 21].

M2 macrophages have regulatory or wound-healing functions [2]. Regulatory functions show anti-inflammatory and phagocytic properties, which are important in the resolving phases of inflammation, producing the immunosuppressive cytokine IL-10, and can be triggered by immune complexes, prostaglandins, apoptotic cells, and IL-10 [31]. On the other hand, wound-healing functions produce IL-4 and positively regulate the activity of the arginase pathway, obtaining ornithine and urea as products from arginine; this enzyme is involved in the production of polyamines and collagen, thus regenerating damaged tissue [32, 33].

Recently, M2 macrophages have been subclassified into subtypes, which leads to more complex systematization: M2a, M2b, and M2c [7]. M2a macrophages are activated by IL-4 and IL-13, leading to upregulated expression of arginase-1 (Arg-1) and CD206, impact presentation by the MHC II system, and production of IL-10 and TGFβ, leading to tissue regeneration and the internalization of proinflammatory molecules to prevent the inflammatory response [34]. M2b macrophages produce IL-1, IL-6, IL-10, and TNFα in response to immune complexes or bacterial LPS, leading to Th2 cell activation and anti-inflammatory activity; however, they can inhibit the polarization of naive macrophages into M1 macrophages, thus promoting the survival of microorganisms or tumor cells [35]. M2c macrophages are activated by IL-10, TGFβ, and glucocorticoids [19]. These cells express CD206 and CD163 surface markers and then produce large amounts of IL-10, TGFβ, and chemokines, leading to suppression of the inflammatory response. These macrophages are involved in tissue homeostasis because of their high phagocytic capacity [34, 36]. Another subtype is M2d macrophages, which respond to IL-6, agonist TLRs and adenosine stimuli [37]. These macrophages are characterized by heterogeneous macrophage populations and pro- and anti-inflammatory functions and are also known as tumor-associated macrophages (TAMs) because of their immunosuppressive capacity and ability to promote angiogenesis in cancer (Table 1) [28].

Although the activation state of M2-type macrophages involves heterogeneous macrophage populations, some markers are shared between subtypes, so the strict division of macrophages into subtypes has not been fully elucidated [35]. Tissues contain a diverse range of stimuli that give rise to mixed populations of M2 macrophages, so these cells are of scientific interest because of their broad spectrum of activation states [6].

## 3. Adipose Tissue Inflammation in Obesity

Obesity is considered a global epidemic that consequently reduces quality of life, presents a lower life expectancy, and increases health care costs [38]. This disease is a risk factor for the development of IR, T2D, hyperlipidemia, CVD, metabolic-associated liver disease (MALD), hyperuricemia, and disorders of the immune system [39, 40]. Obesity is a multifactorial chronic disease that cannot be considered only the result of an energy imbalance between caloric intake and expenditure; however, a series of metabolic abnormalities, oxidative stress, mitochondrial and immunological dysfunction, and chronic inflammation in obese individuals have also been identified [10, 41]. Although AT is well known to generate an immune response to the excessive presence of nutrients, there is still no knowledge about the initial inflammatory trigger involved [42]. Therefore, more attention is currently being devoted to the study of endogenous anti-inflammatory mediators and other important therapeutic targets in AT inflammation.

AT, commonly known as body fat, is a complex loose connective tissue composed of mature adipocytes, which are the cells responsible for fat storage; preadipocytes; fibroblasts; mesenchymal cells; vascular endothelial cells; pericytes; the stromal vascular fraction (SVF); immune cells, including macrophages and T cells; and the supportive matrix of extracellular proteins [43, 44]. Its functions include storing energy in the form of lipids and protecting and insulating vital organs of the body [45]. This tissue has endocrine activity and secretes various bioactive molecules, including adipokines, cytokines, and hormones, such as leptin, adiponectin, resistin, estradiol, and TNFα [46, 47]. There are two types of AT: white adipose tissue (WAT) and brown adipose tissue (BAT). WAT is composed of 5%–50% of the body weight, stores excess energy in the form of triglycerides and plays an important role in endocrine signaling by releasing hormones. WAT can be divided into subcutaneous adipose tissue (SAT) and visceral adipose tissue (VAT). SAT acts as a thermal insulator and protector, whereas VAT is more related to metabolic processes. BAT is located in the neck and large blood vessels of the thorax, plays an important role in adaptive thermogenesis and decreases in size with age [48, 49].

Adipocytes, the main cells present in the AT, can develop through two processes: hypertrophy, described as an increase in cell size, and hyperplasia, which is the increase in the number of cells from a precursor cell—preadipocyte—that manage to differentiate until its final stage, becoming a mature adipocyte [50]. Both processes of adipocyte development occur under healthy conditions; adipocytes begin to grow at a certain time, increasing their fat volume, and when they reach the critical threshold in size, the hyperplasia process occurs; the hypertrophied cell stimulates a preadipocyte, thus generating a new adipose cell [51].

During the development of adipocytes by hypertrophy, a transient inflammatory state occurs, which is considered necessary to carry out this process [50]. The problem arises when in a state of obesity, this situation is continuous, since a larger size of the adipose cell together with the concomitant inflammation compromises the integrity and functionality of the adipocyte by being excessively hypertrophied, which modifies the metabolic behavior of the cell, generating alterations in the tissue and even triggering the apoptosis of the adipocytes [52]. Under these circumstances, obese AT has an altered secretory profile with greater production of leptin and less adiponectin, lower sensitivity to insulin in tissues, mitochondrial dysfunction, and greater stress in the endoplasmic reticulum; these disruptions increase basal lipolysis and decrease de novo lipogenesis, which results in alterations in the cellular structure of adipocytes [53].

In healthy individuals, adipocytes help maintain an adequate energy balance and a normal body temperature [54]. Lipids are predominantly stored in the SAT, but in obese individuals, the SAT can reach its expansion limit or cannot expand adequately to store excess energy [54]. When adipocytes reach their triglyceride storage limit, they begin to be deposited ectopically in other tissues involved in metabolic homeostasis, that is, skeletal muscle, liver and VAT; this is due to the increase in basal lipolysis, a process known as the *“*overflow hypothesis,*”* thus altering the metabolic balance, which is associated with the development of chronic inflammation and lipotoxicity [55, 56]. The increase in the flow of free fatty acids and the release of inflammatory factors act together as triggers of IR and local inflammation to later cause systemic IR and metaflammation [57]. Furthermore, BAT is often reduced under obesogenic conditions, decreasing the body's ability to burn calories and increasing the risk of metabolic diseases [58]. Overall, the hypertrophy capacity of SAT and fat storage in VAT are critical factors in the development of obesity, IR, and their associated complications [59].

### 3.1. Macrophages in Healthy Adipose Tissue

The microenvironment of healthy AT is a complex and dynamic system that plays a crucial role in maintaining energy balance and metabolic homeostasis [60]. In a healthy state, the cells that compose this tissue interact in a balanced manner, promoting an anti-inflammatory and metabolically active environment [61]. The AT microenvironment is characterized by a well-regulated balance in the secretion of adipokines, cytokines, and hormones, which are essential for normal metabolic processes such as glucose and lipid metabolism, insulin sensitivity, and immune responses [62]. These adipokines, such as leptin and adiponectin, are responsible for regulating appetite, increasing insulin sensitivity, and regulating systemic inflammation [63]. Adiponectin has anti-inflammatory effects and promotes insulin sensitivity, helping to maintain efficient regulation of metabolism [64]. Furthermore, the vascular network within the AT is crucial for nutrient and oxygen supply, supporting the high metabolic activity of adipocytes [65].

In healthy AT, this vasculature is well developed, allowing for efficient transport of lipids and glucose into adipocytes and facilitating the removal of waste products [44]. Additionally, the presence of immune cells, such as macrophages, in healthy AT is tightly regulated by cytokines and transcription factors [66]. These immune cells play a role in tissue remodeling and maintaining a balance between proinflammatory and anti-inflammatory signals, which is critical for preventing chronic inflammation and associated metabolic disorders [66, 67]. Anti-inflammatory cytokines such as IL-10 and TGFβ predominate, maintaining controlled immune activity [68]. Macrophages in the AT of healthy individuals are usually of the M2 type, which promotes tissue repair and the resolution of inflammation [69].

Furthermore, transcription factors such as PPARγ and CCAAT/enhancer binding protein α/β (C/EBPα/β) are essential for adipocyte differentiation and lipid metabolism;for example, PPARγ plays a key role in the formation of new adipocytes and in regulating insulin sensitivity [70, 71]. On the other hand, the extracellular matrix (ECM), an important factor in healthy AT, provides structural support and regulates cell signaling, which influences the cell differentiation, proliferation, and survival of resident cells [72]. The ECM components are constantly remodeled to adapt to changes in size and function required by adipocytes, especially during periods of weight gain or weight loss [73]. A healthy ECM ensures that AT remains flexible and capable of expanding or contracting as needed without triggering fibrosis or other pathological changes [74].

Overall, the microenvironment of healthy AT is finely tuned by a network of cellular and molecular signals that support metabolic health, where the tissue can respond to nutritional changes and interact with other organs through endocrine signaling [75]. Disruptions to this microenvironment, such as excessive adipocyte hypertrophy, chronic inflammation, or impaired angiogenesis, can lead to metabolic diseases or initiate metabolic syndrome [76]. Therefore, maintaining the integrity of the AT microenvironment is essential for overall health and well-being [75].

### 3.2. Macrophages in Obese Adipose Tissue

Obesity creates a unique AT microenvironment characterized by chronic inflammation and altered immune responses [76]. This environment plays a key role in metabolic dysfunction and can contribute to several metabolic health issues, including IR, T2D, and cancer [77]. AT has a high capacity to adapt to excess energy intake, and its expansion through hypertrophy and hyperplasia contributes to the development of a chronic low-grade inflammatory state, also known as metaflammation [78]. Excess lipid storage leads to adipocyte hypertrophy, which in turn results in hypoxia, cellular stress, and dysfunction. TLR2 is activated by free fatty acids, triggering an inflammatory response through adipocyte hypertrophy that induces both adipocytes and resident macrophages to secrete proinflammatory cytokines such as TNFα, IL-6, IL-1β, and monocyte chemotactic protein-1 ([MCP-1], also known as CCL-2). These cytokines promote the infiltration of immune cells, primarily macrophages [79–81]. As macrophages accumulate in response to cellular stress, they undergo a phenotypic switch toward the proinflammatory M1 phenotype, further amplifying proinflammatory cytokine production and immune cell recruitment. The release of these inflammatory mediators into the bloodstream can affect other tissues, perpetuating a cycle of systemic inflammation [10, 82, 83].

In addition to lymphocytes, foam cells, mast cells, eosinophils, and neutrophils, macrophages dynamically infiltrate AT during the development of obesity and IR, especially under high-fat diet conditions [84, 85]. These immune cells secrete inflammatory factors that alter the AT microenvironment, contributing to the shift from an anti-inflammatory state to an inflammatory state [85]. Macrophages, referred to as AT-associated macrophages (ATMs), are key regulators of inflammation and metabolic homeostasis within ATs and play roles in regulating immune responses and mitochondrial function in adipocytes [48, 86]. ATMs originate from circulating monocytes recruited by MCP-1 and differentiate within both VAT and SAT, with the capacity to self-renew locally [48, 87].

Additionally, adipokines play crucial roles in obesity-related complications [88]. Lipo-inflammation in AT is linked to an imbalance in the adipokine profile, notably with increased leptin and decreased adiponectin levels [89–92]. Leptin has immunomodulatory functions, whereas adiponectin acts as an insulin sensitizer and has anti-inflammatory effects [91]. This altered secretory profile may contribute to the metabolic dysregulation observed in obesity [93]. Furthermore, changes in the ECM structure and reduced angiogenic capacity in AT are involved in its pathological expansion, leading to fibrosis and hypoxia, which further impair adipocyte function and aggravate inflammation [73, 94].

Adipocytes with limited hyperplastic potential undergo hypertrophy and exacerbate metaflammation, leading to reduced lipolysis, an impaired insulin response in metabolic tissues, and diminished glucose uptake—key factors in the development of IR and glucose intolerance [95, 96]. As a result, the VAT becomes the primary triglyceride storage site when the SAT fails to store excess energy effectively [97]. These alterations in the obese AT microenvironment and increased visceral fat deposition constitute major risk factors for metabolic syndrome, IR, T2D, CVD, and certain cancers [98, 99].

Understanding the mechanisms linking lipo-inflammation to obesity-associated pathologies is essential for developing targeted therapeutic strategies [100]. Additionally, sex-specific fat distribution patterns partly explain why women—who tend to accumulate fat in the gluteal–femoral region—have a lower incidence of cardiovascular events than men do, who accumulate fat more centrally [101].

#### 3.2.1. Impact of the Obese AT Microenvironment on Macrophage Polarization

The phenotype, abundance, and activation state of ATMs are recognized as key modulators in the development of obesity-associated metabolic risk. In lean AT, ATMs acquire the M2 phenotype, promoting an anti-inflammatory response and tissue repair in AT [86]. The main function of M2 macrophages in this context is to regulate systemic glucose homeostasis by inhibiting preadipocyte proliferation through the CD206/TGFβ signaling pathway, which induces IL-10 release [102, 103]. Additionally, IL-25 stimulates M2 macrophage polarization and enhances their interaction with adipocytes, promoting energy metabolism, improving mitochondrial function in adipocytes, and regulating lipid accumulation in WAT, VAT, and the liver [104]. However, under conditions of excess energy and obesity, ATMs polarize toward the M1 phenotype. M1 macrophages play key roles in initiating and maintaining the inflammatory state by releasing cytokines that attract circulating monocytes through chemoattractants. This results in increased macrophage accumulation and the development of chronic low-grade inflammation. This inflammatory state not only affects AT function but also contributes to systemic metabolic dysfunction [105, 106].

Mechanistically, inositol-requiring enzyme 1α (IRE1α) has been identified as an important protein in the development of obesity and metabolic syndrome. Its activation by obesogenic factors—such as stearic, palmitic, and myristic fatty acids and LPS—reduces the population of M2 ATMs and increases the number of M1 ATMs. Moreover, the IRE1α pathway negatively affects BAT activity and prevents WAT browning [107, 108]. Similarly, the inhibition of key regulators of browning and homeostasis, such as fibroblast growth factor 21 (FGF21) and NAD-dependent sirtuin-1 deacetylase (SIRT1), increases miR-34a levels in obese AT [48, 109]. miR-34a is released into ATMs via lipid-rich exosomes from hypertrophic adipocytes and reduces the expression of KLF4, a transcription factor essential for M2 polarization. These exosomes also carry miR-155, which promotes M1 polarization [110, 111]. In contrast, preadipocyte-derived SVF exosomes have been shown to transactivate Arg-1 by inhibiting miR-34a and miR-155, thus promoting M2 polarization and contributing to preadipocyte hyperplasia and AT browning [48, 112]. On the other hand, chronic hyperglycemia activates macrophages through the JNK and ERK pathways, further promoting monocyte infiltration and differentiation into M1 macrophages [113]. Notably, increased macrophage infiltration into AT has been positively correlated with obesity development in both human and mouse models [82, 114, 115], suggesting that ATMs are among the most important immune cells involved in adipose tissue expansion and metabolic‒immune interactions in obesity.

#### 3.2.2. Hypoxia is the Main Driver of M1 AT Macrophages and AT Dysfunction in Obesity

Hypoxia in obesity is a key factor driving metabolic dysfunction, macrophage polarization, and chronic inflammation in AT. As AT expands in response to excessive caloric intake, the increasing distance between adipocytes and the vascular network does not increase proportionally, leading to inadequate oxygen delivery, particularly in hypertrophic adipocytes [62]. This hypoxic environment impairs proteasomal degradation of HIF-1α, stabilizing this transcription factor in adipocytes and macrophages through the IGF receptor-activated PI3K‒Akt pathway [116]. The resulting activation of HIF-1α initiates a cascade of proinflammatory and metabolic changes, playing a central role in the recruitment and polarization of macrophages toward the M1 phenotype, which exacerbates local and systemic inflammation [117].

By binding to its coactivators p300/CBP, HIF-1α upregulates genes involved in glycolysis, angiogenesis, and inflammation, including glucose transporter 1 (GLUT1), VEGF, and NF-κB. These genes not only support AT survival under low oxygen but also contribute to immune dysregulation [116]. For example, HIF-1α-induced GLUT1 expression promotes basal glucose uptake in a noninsulin-dependent manner, enhancing glycolytic metabolism—a hallmark of both hypoxic adipocytes and proinflammatory M1 macrophages [118]. This phenomenon, known as the “Warburg effect,” reflects a metabolic shift from oxidative phosphorylation to anaerobic glycolysis, supporting the energy demands of activated immune cells. Thus, GLUT1 overexpression in hypoxic AT facilitates metabolic reprograming of macrophages toward the M1 phenotype [119].

Moreover, IR induced by chronic HIF-1α activity impairs GLUT4 function and translocation, reducing insulin-mediated glucose uptake in adipocytes and skeletal muscle [120]. This dysfunction further contributes to systemic hyperglycemia and fuels inflammation, reinforcing a vicious cycle: hypoxia promotes HIF-1α activation, which enhances GLUT1 expression and M1 macrophage activation, whereas IR impairs GLUT4 function and glucose homeostasis. The contrasting roles of GLUT1 and GLUT4 illustrate how metabolic shifts in obesity are tightly linked to macrophage polarization [121].

VEGF, another target gene of HIF-1α, plays a critical role in regulating angiogenesis in response to increased metabolic demands during AT expansion [122]. However, in obesity, persistent HIF-1α activation leads to excessive and disorganized angiogenesis, which is insufficient to resolve hypoxia. This abnormal vascularization promotes chronic inflammation, fibrosis, and further recruitment of immune cells, particularly M1 macrophages. VEGF transcription is also modulated by STAT3, PCG-1, and PPARγ, and its dysregulation amplifies AT inflammation. Therefore, the VEGF-HIF-1α axis contributes not only to vascular dysfunction but also to immune cell infiltration and M1 macrophage-mediated inflammation [123, 124].

In addition, the platelet-derived growth factor receptor (PDGFR) pathway, particularly under hypoxic conditions, has emerged as a key regulator of AT remodeling and fibrosis. PDGFRα and PDGFRβ are expressed in adipose progenitor and stromal cells, and their downstream signaling is enhanced by HIF-1α [125]. PDGFRα supports adipogenesis, whereas PDGFRβ+ pathways under hypoxia inhibit PPARγ, limiting adipocyte differentiation and promoting fibrosis. This also stimulates the proliferation of fibroblasts and enhances ECM deposition, reducing AT flexibility and expanding capacity. Importantly, increased PDGFR signaling also enhances macrophage recruitment and polarization to the M1 phenotype, further sustaining AT inflammation [125, 126]. Notably, inhibition of HIF-1α in PDGFRβ+ preadipocytes was shown to restore adipogenesis and improve glucose tolerance in a PPARγ-dependent manner, suggesting that targeting the PDGFR/HIF-1α axis could mitigate both fibrosis and the M1 phenotype in obese AT [127].

The hypoxia-driven impairment of PPARγ expression in both adipocytes and macrophages further reinforces M1 polarization. PPARγ, a nuclear receptor essential for adipogenesis and lipid metabolism, is downregulated under hypoxic and inflammatory conditions, weakening the M2 anti-inflammatory phenotype and favoring M1 activation [127, 128]. This contributes to defective tissue repair and immune resolution. Moreover, the chronic inflammatory state in obese AT leads to the formation of crown-like structures (CLSs), which are composed of proinflammatory macrophages encircling dead or dying adipocytes. These CLSs act as local hubs of inflammation, perpetuating tissue damage, and are strongly associated with IR and metabolic disease progression [129].

#### 3.2.3. The Role of PPARγ in M2 Macrophage Polarization

PPARγ is an important transcription factor in AT that is involved in adipocyte lipid metabolism and anti-inflammatory responses [130, 131]. PPARγ-regulated metabolic adaptation leads to subcutaneous and brown fat deposition in adipocytes, reduced circulating lipids, and decreased lipotoxicity [127]. Alterations in PPARγ expression decrease fatty acid uptake and change the metabolic programing of adipocytes and ATMs [128]. The presence of lipid molecules plays a role in macrophage polarization and is influenced by the PPARγ signaling pathway [127, 132]. This regulatory pathway promotes the anti-inflammatory M2 metabolic program and inhibits M1 polarization [133]. The metabolic features displayed by the PPARγ-regulated ATM phenotype include increased amino acid metabolism by Arg-1, inhibition of glycolysis, increased lipolysis, elevated β-oxidation of fatty acids, and increased mitochondrial biogenesis [134]. Studies have shown that elevated IL-4 induces the expression of fatty acid translocase (FAT) (CD36), a receptor that transports long-chain fatty acids and oxidizes low-density lipoproteins (ox-LDLs); moreover, IL-4 further increases PPARγ expression [135]. Therefore, fatty acid metabolism and PPARγ expression are necessary for anti-inflammatory ATM polarization [128]. The inhibition of PPARγ in ATMs of obese individuals induces an M1 proinflammatory metabolic program via IFNγ, which is characterized by increased aerobic glycolysis and fatty acid synthesis, decreased lipid transport, and amino acid metabolism that is regulated by iNOS and generates a greater amount of ROS [132, 136].

In recent years, the role of mammalian target of rapamycin (mTOR) in relation to PPARγ in the metabolic program of adipocytes and macrophages has been studied [137]. Induction of mTOR in AT is associated with adipocyte hypertrophy, but its inhibition results in a decrease in the size and number of adipose cells, resulting in probable protection against obesity [137, 138]. However, the inhibition of mTOR, despite decreasing lipid uptake and storage, results in hypertriglyceridemia, which is why mTOR and PPARγ also modulate lipid metabolism in the plasma [138].

In obesity, under conditions of excess energy, mTOR enhances the transcriptional activity of PPARγ and increases the synthesis of proteins involved in lipid metabolism, such as lipoprotein lipase (LPL) and glycerol kinase (GyK) [139]. Therefore, mTOR activation is important for maintaining plasma lipid homeostasis and increasing lipid uptake and storage by activating PPARγ [140]. mTOR acts on PPARγ depending on metabolic and nutritional status to execute metabolic responses (e.g., synthesis of cholesterol, fatty acids, and proteins involved in lipid metabolism) and responses associated with M1 ATMs [141].

Recently, semaphorin-6D (Sema6D) was shown to link the mTOR and PPARγ pathways in ATM polarization [142]. Sema6D, acting as a receptor (reverse signaling), is activated by mTOR and binds to the protein kinase C-Abl; this induces the transcription of PPARγ, allowing polarization toward M2 anti-inflammatory macrophages and the regulation of fatty acid metabolism. c-Abl also activates the differentiation of preadipocytes (hyperplasia) [142, 143]. The inhibition of mTOR or Sema6D decreases the expression and activity of PPARγ; therefore, the polarization of ATMs toward M2 is affected, their metabolic programing and lipid uptake decrease, the expression of CD36 is also affected, and M1 polarization is improved [135].

Adiponectin, a plasma protein secreted by adipocytes, protects the body against CVD and metabolic diseases [144]. There is also a regulatory cycle between PPARγ and adiponectin; the increase in adiponectin available in the blood is associated with greater activation of PPARγ, and this factor further stimulates the synthesis and release of bioavailable adiponectin [145]. A study revealed that genetically modified mice that overexpress adiponectin present with hypotriglyceridemia and that lipids are stored in the SAT [146]. On the other hand, this adipokine also exerts a regulatory effect on the polarization of macrophages toward the M2 phenotype [147]. Animal studies have revealed that modified mice that do not express adiponectin exhibit increased production of the inflammatory cytokines TNFα, IL-6, and MCP-1 in ATMs, whereas the number of M2 macrophages and their markers, such as Arg-1 and IL-10, decreases [148]. In addition, another study reported that the levels of markers and polarization of M2 macrophages increase and that the level of ROS and polarization toward the M1 phenotype decreased in macrophages treated with recombinant adiponectin [149]. Stimulation of adiponectin expression decreases the progression of obesity and obesity-associated metabolic abnormalities by promoting the resolution of the low-grade inflammatory state in AT [150].

Studies related to microRNAs have been very important and useful in recent years. One known regulator of inflammation and IR is miR-223 [151]. The expression of miR-223 is regulated downstream by PPARγ; consequently, the PPARγ/miR-223 axis regulates the polarization of macrophages toward an anti-inflammatory M2 phenotype to maintain homeostasis in AT and improve insulin sensitivity [152, 153]. Investigating the mechanisms by which mTOR-Sema6D and adiponectin modulate PPARγ and by which PPARγ regulates miR-223 expression may be highly important for the development of therapeutic approaches related to PPARγ activity, safe lipid storage, ATM polarization, and metabolic reprograming of adipocytes and macrophages [154].

### 3.3. Macrophages in CLSs in Obese AT

**Figure 3:**
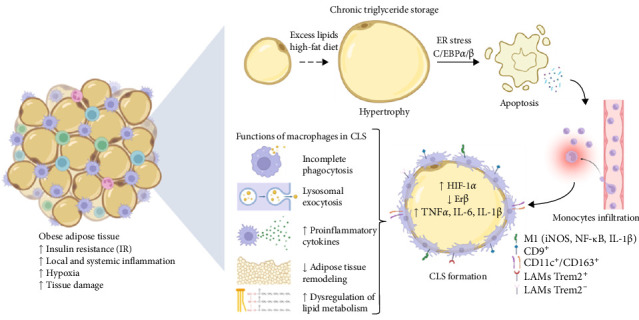
Formation of crown-like structures (CLSs) in obese adipose tissue: macrophage subtypes and metabolic consequences. In obesity, hypertrophied and dying adipocytes are surrounded by macrophages that form CLSs. These structures are in a hypoxic microenvironment and are composed mainly of proinflammatory M1 macrophages (including the CD9^+^ and CD11c^+^ subsets) but also include transitional subtypes (e.g., CD11c^+^/CD163^+^) and LAMs expressing Trem2^+^. CLS formation contributes to local hypoxia, chronic inflammation, impaired lipid metabolism, and insulin resistance.

ATMs exhibit high proliferative capacity and decreased apoptosis in the early stages of WAT hypertrophy following a high-fat diet [155]. However, continuous intake of a high-fat diet leads to the development of glucose intolerance and IR because adipocytes upregulate the endoplasmic reticulum stress pathway C/EBPα/β, resulting in an altered WAT microenvironment [156, 157]. Alterations and stress in the microenvironment and elevated lipid storage in adipocytes lead to adipose cell death, and cell debris is removed by ATMs. The large size and volume of apoptotic adipocytes cannot be phagocytosed and engulfed by a single macrophage, and as a result, monocytes infiltrate and subsequently differentiate into M1 macrophages, which aggregate around adipose cells, form a crown-shaped structure, and eliminate dead adipocytes via lysosomal exocytosis, a hallmark of AT remodeling in obesity [158]. As alterations in adipocyte function and stress progress, lysosomal exocytosis abruptly decreases, and excessive lipid accumulation results in increased tissue damage in AT (Figure 3).

CLS is a primary/key histological feature in the inflammatory and apoptotic process of AT. The dominant macrophages in CLSs are of the M1 phenotype and release proinflammatory factors (NF-κB, iNOS, IL-1β, IL-6, and TNFα) and free fatty acids, which contribute to increased hyperinsulinemia and IR. Furthermore, ATMs of the M1 phenotype activate the transcription factor HIF-1α; as a result, these hypoxic ATMs agglomerate to form CLSs and express elevated iNOS and IL-1β as obesity progresses [2, 129]. These CLSs are the main site of hypoxia and local inflammation in the later stages of obesity. Recently, estrogen receptor beta (ERβ) was shown to be key for the initiation of CLS formation by acting on HIF-1α. A recent study confirmed that CLS formation in SAT and VAT is increased in obese ERβ^−/−^ mice. Obese mice treated with an ERβ agonist show decreased CLS formation in SAT and VAT, as well as decreased HIF-1α activation. Stimulating ERβ in macrophages that compose CLSs and infiltrating macrophages may help reduce the number of CLSs, decreasing the degree of IR in Ats [129]. Specifically, targeting the hypoxic microenvironment, hypoxia-driven signaling and M1 ATM differentiation in CLSs may offer therapeutic potential for improving metabolic outcomes in obese individuals.

Tetraspanin-29 (CD9) macrophages represent a distinct ATM population and have gained attention for their role in AT metabolic dysfunction. They are cells that, owing to the expression of the surface marker CD9, can form CLSs around dead or dying adipocytes, thus playing a role in the removal of apoptotic adipocytes; they also present increased lipid accumulation through exosome formation, are metabolically active, and are responsible for amplifying inflammatory signaling in obese Ats [159]. CD9^+^ ATMs coexpress elevated levels of the surface markers CD11c, CD16, and CD206, which express inflammatory transcription factors such as AP-1 and NF-κB, and these induce the release of the proinflammatory mediators IL-6, IL-18, and TNFα, which promote IR and exacerbate AT dysfunction [114]. Research has shown that CD9^+^ ATMs express genes involved in lipid handling and fibrosis, further implicating them in AT remodeling and metabolic complications.

On the other hand, other subpopulations of CD11c^+^/CD163^+^ ATMs are part of the CLS in VAT and SAT and have special relevance because of their dual or transitional role in regulating the local inflammatory and metabolic environment and in processes of remodeling or resolution of inflammation, which adds complexity to the immune response in obesity [160]. CD11c^+^/CD163^+^ macrophages actively participate in the response to adipocyte death and in the regulation of inflammation in expanded AT. CD11c expression is related to the proinflammatory M1 profile, while CD163 expression could be involved in the modulation of inflammation and protection against excessive tissue damage due to its anti-inflammatory M2 profile [161]. Targeting CD9^+^ macrophages as well as hybrid M1/M2 macrophage subtypes could offer a novel therapeutic approach to reduce inflammation and improve metabolic outcomes in obese individuals.

Recently, a subpopulation of macrophages known as lipid-associated macrophages (LAMs) was discovered within the CLS; these macrophages have the main functions of lipid catabolism and modulate the inflammatory response of the AT through the loss of homeostasis and death of hypertrophic adipocytes [162]. The LAM that forms the CLS are the only immune cells in obese AT that express lipid receptor trigger receptor expressed on myeloid cells 2 (Trem2) [163]. Studies have shown that LAM-Trem2^−^ increases hypertrophy in adipocytes and leads to hypercholesterolemia, inflammation, and IR; therefore, metabolism in obese individuals is further impaired [164, 165]. However, LAM-Trem2^+^ CLSs formed in obese adipocytes can locally contain lipid droplets, decrease AT inflammation and hypertrophy, and prevent the loss of lipid homeostasis at the systemic level, since the transcriptional profile triggered by Trem2 induces the expression of mediators involved in lipid handling, such as CD36 and PLIN2 [164, 166]. These findings suggest that the downstream pathway of Trem2 is activated by recognition signals, indicating the loss of homeostasis in adipocytes and lipid accumulation in the ECM [167]. Although CLS formation has been reported to be positively correlated with M1 macrophages, increased severity of obesity and metabolic syndrome, these studies identify Trem2^+^ CLSs as a new potential therapeutic target in obesity.

## 4. ATMs and Their Interactions Within the Lean and Obese AT Microenvironments

AT is a highly dynamic and heterogeneous organ composed not only of adipocytes and ATMs but also of a diverse array of other resident cell types that interact within a complex microenvironment. These interactions are fundamental to maintaining tissue homeostasis and regulating systemic metabolic processes [168, 169]. ATMs play a central role as regulators of immunometabolic balance. Under lean, healthy conditions, ATMs engage in close crosstalk with adipocytes by clearing apoptotic cells and excess lipids, and by responding to adipokines such as adiponectin and leptin, which modulate their activation state and function [170]. Importantly, adipocytes themselves are not passive lipid storage units; rather, they act as active immunometabolic cells capable of modulating ATM phenotypes through the secretion of free fatty acids and proinflammatory cytokines, thereby maintaining a balance between inflammatory and anti-inflammatory processes [90].

To fully understand how macrophage phenotypes influence AT function and systemic metabolic health, it is essential to consider their interactions with other resident cells in the adipose tissue microenvironment. Crosstalk between ATMs, adipocytes, endothelial cells, and T lymphocytes plays a central role in maintaining tissue homeostasis and shaping the local immune landscape [171]. Adipocytes release adipokines, lipids, and chemotactic signals that modulate macrophage recruitment and polarization [88]. Endothelial cells regulate immune cell infiltration and respond to hypoxia by expressing adhesion molecules and inflammatory mediators [172]. Moreover, T lymphocytes, particularly CD4^+^ T cells and regulatory T cells (Tregs), influence the inflammatory tone through cytokine production and direct interactions with ATMs [173]. These complex cell–cell interactions are crucial for modulating ATM function, which in turn affects AT remodeling, lipid storage, and systemic metabolic regulation.

Furthermore, the SVF of AT comprises a variety of immune and structural cells that profoundly shape ATM function. Cytotoxic CD8^+^ T cells and Th1 subsets promote the polarization of proinflammatory M1-like macrophages, whereas Th2 cells and Tregs facilitate the development of anti-inflammatory M2-like macrophages [174, 175]. B cells and natural killer (NK) cells contribute to the inflammatory milieu through antibody production and cytotoxic mechanisms, respectively [176, 177]. Additionally, eosinophils and type 2 innate lymphoid cells (ILC2s) support the maintenance and function of M2 ATMs via the IL-4 and IL-13 signaling pathways in lean AT [178]. Structural cells such as fibroblasts and pericytes participate in ECM remodeling and fibrosis, which significantly modulates ATM activity, particularly in the context of obesity-induced tissue remodeling [73]. Moreover, mesenchymal stem cells and preadipocytes secrete a variety of factors that influence macrophage recruitment and polarization [174]. This intricate network of cellular interactions is essential not only for regulating local inflammation but also for preserving the remodeling capacity and metabolic functions of AT. Disruption of this cellular crosstalk in obesity results in chronic inflammation, adipose tissue dysfunction, and systemic metabolic derangements such as IR [179].

## 5. Obesity-Associated Metabolic Risk and Macrophage Phenotypic Switch

Obesity-related inflammation is a critical factor in the development of several chronic diseases, including T2D, CVD, MALD, and certain cancers [50, 84]. A key player in this type of inflammation is macrophages, whose transcriptomic/proteomic and phenotypic plasticity allows them to adapt to different tissue environments. In lean AT, macrophages predominantly exhibit an M2-like phenotype, which supports tissue remodeling, insulin sensitivity, and immune homeostasis. However, in obesity, there is a shift toward a proinflammatory M1-like ATM phenotype characterized by the production of cytokines such as TNFα and IL-6, which disrupt insulin signaling and promote systemic IR [180].

This phenotypic switch contributes to the progression of T2D, as M1 macrophages accumulate in VAT and exacerbate local and systemic inflammation. Similarly, M1 macrophage infiltration in vascular tissues promotes atherosclerosis, linking inflammation to CVDs [181]. In the liver, Kupffer cells (liver-resident macrophages) also adopt a proinflammatory phenotype in response to lipid overload, facilitating the progression from simple steatosis to MALD and fibrosis [182]. Furthermore, chronic inflammation mediated by M1 macrophages creates a microenvironment that supports tumorigenesis in obesity-related cancers such as breast and colorectal cancer [158].

Interestingly, not all inflammation is detrimental. Recent studies suggest that controlled proinflammatory signaling is necessary for healthy AT remodeling and expansion. For example, transient activation of inflammatory pathways in the SAT may promote safe lipid storage and prevent ectopic fat deposition [183, 184]. Thus, the balance between M1 and M2 macrophage phenotypes in different AT depots could determine whether inflammation leads to metabolic dysfunction or adaptation in response to excess energy. Understanding the dynamic role of macrophage phenotypes in obesity-induced inflammation provides valuable insights into metabolic disease pathogenesis and could lead to the development of new therapeutics that target ATM plasticity.

## 6. Concluding Remarks

The phenotypic switch of macrophages in the obese adipose microenvironment has become a critical therapeutic target for addressing obesity-associated metabolic risk related to AT inflammation and metabolic dysfunction. Recent advances in understanding the underlying mechanisms of this switch, including the role of hypoxia, lipid signaling, and the interplay of cytokines and adipokines, have paved the way for innovative therapeutic strategies. Emerging approaches include the use of small molecules and biologics aimed at modulating key signaling pathways, such as inhibitors of NF-κB and HIF-1α or activators of PPARγ and STAT6, to promote the anti-inflammatory M2 phenotype. Additionally, nanoparticle-based drug delivery systems are being developed to selectively target macrophages within obese adipose tissue, offering precision in reprograming their polarization without affecting systemic immunity. Immunotherapy approaches, such as adoptive transfer of engineered macrophages or the use of monoclonal antibodies against proinflammatory cytokines such as TNF-α and IL-6, also show promise. Another exciting avenue is the application of metabolic reprograming agents, which aim to shift macrophage metabolism from glycolysis (associated with M1 polarization) to oxidative phosphorylation (favoring M2 polarization). Furthermore, therapies targeting the gut‒adipose axis, such as prebiotics and probiotics, are being explored to indirectly influence macrophage function by modulating AT and systemic inflammation and lipid metabolism. While these novel therapies show significant potential, challenges remain in optimizing their safety, efficacy, and delivery, emphasizing the need for continued research and clinical trials to bring these approaches closer to widespread application in managing obesity and its associated diseases.

## Figures and Tables

**Table 1:** Stimuli, surface markers, secreted cytokines, and biological functions of different types of macrophages.

Phenotype	Stimuli	Surface markers	Expressed mediators	Functions	References
M1	LPS IFNγ TNFα RNS/ROS	CD80, CD86, TLR2, TLR4, HLA-DR, IFNγR	TNFα, IL-1β^high^, IL-6, IL-12^high^, IL-1α, IL-23, IL-18, CXCL9, CXCL10, CXCL11, CCL5, CCR7, CXCL16, GM-CSF Arg-2, iNOS	Proinflammatory Th1 response antitumor capacity	[2, 20, 21]
M2a	IL-4, IL-13	CD163, CD206, Arg-1	IL-10, TGFβ, CCL17, CCL24, CSF-1	Anti-inflammatory tissue remodeling	[15, 35]
M2b	IL-1β, TLR ligands, immune complex	CD86	TNFα, IL-1β, IL-6 IL-10, CCL1	Th2 activation, pro- and anti-inflammatory immunoregulation	[15, 36]
M2c	Glucocorticoids, IL-10, TGFβ	CD206, CD163, Arg-1	IL-10, CCL18, CXCL13, TGFβ, CCL16	Anti-inflammatory phagocytic and apoptotic capacity	[35, 37]
M2d	IL-6, adenosine receptor ligands, TLR ligands	VEGF, ILT3R	IL-10, CCL18, CCL22, VEGF	Pro- and anti-inflammatory angiogenesis, immunosuppressive tumor progression	[20, 29]

*Note:* Arg-1, arginase type-1; Arg-2, arginase type-2; CSF-1, colony stimulating factor 1; ILT3R, immunoglobulin-like transcript 3.

## Data Availability

No underlying data were collected or produced in this study.
